# Cancer immunotherapy in routine cost‐effective cancer care?

**DOI:** 10.15252/emmm.201809660

**Published:** 2018-10-15

**Authors:** Sir Marc Feldmann

**Affiliations:** ^1^ Nuffield Department of Orthopaedics, Rheumatology and Musculoskeletal Sciences University of Oxford Oxford UK

**Keywords:** Cancer, Immunology

## Abstract

Therapy‐triggered autoimmunity and inflammation in many cancer patients, as well as treatment costs, hamper the success of cancer immunotherapy. The study by Pfeiffer *et al* in this issue of *EMBO Molecular Medicine* constitutes a clear step towards a more simple, scalable and affordable approach in generating *in situ* T cells reprogrammed with chimeric antigen receptors (CARs) (Pfeiffer *et al*, 2018).

The existence of immune responses to human cancer is nowadays well established (reviewed in Miller & Sadelain, 2015), and immunotherapy has become an increasingly important part of cancer medical care. The first real therapeutic success of immunotherapy in cancer was the use of antibodies targeted to cancer cells, which harnessed the immune response to help deliver their therapeutic effect, such as rituximab (anti‐CD20) based on Fc engagement (Mellor *et al*, 2013), or trastuzumab (anti‐HER2) (Carter *et al*, 1992) based on FcR (and possibly other mechanisms). For many however, “cancer immunotherapy” evokes “checkpoint inhibitors”, i.e., antibodies to inhibitory molecules. Successful clinical examples are CTLA‐4 (Leach *et al*, 1996), PD‐1 and PD‐L1 (Topalian *et al*, 2012), but other candidates are currently in clinical development. The response is unfortunately not universal, with ~20% of patients exhibiting good responses in susceptible cancers. Moreover, “unleashing” the immune response comes at a price, i.e., autoimmunity and inflammation in many patients, which can at times be life‐threatening and expensive to treat.

Twenty‐five years ago, Zelig Eshhar and his colleagues engineered and expressed chimeric antigen receptors (CAR) constituted of the single Fv chain of the antibody molecule combined with the constant region of the T‐cell receptor, in order to kill tumour cells (Eshhar *et al*, 1993). This pioneering work has been optimized and amplified, and brought to experimental and clinical fruition by several groups (reviewed in Miller & Sadelain, 2015).

For B‐cell tumours expressing CD19, the success of CAR‐T cells has been truly remarkable, and suggests that many more cancers will be treatable, or even curable. However, generating these cells in the scale needed has been a technical feat, to which many companies contributed, and the first product was put on the market, initially at $495,000. This initial success is expected to move on to many other target cancer antigens, and to many patients who require treatment, raising the issue of the financial burden for patients/healthcare systems.

Might there be cheaper alternatives? Yes, the knowledge gained from the clinical success of CAR‐T cells has enabled others to try to simplify and mimic the process, without the costly patient‐specific therapy or extra‐corporeal engineering. The approach reported in this issue of *EMBO Molecular Medicine* (Pfeiffer *et al*, 2018) involves the generation of anti‐viral vectors that target CD8 T cells and can lead to the generation of CAR‐T cells *in vivo*. The reprogramming of human cytotoxic T cells has been achieved using vectors reported recently, which can selectively deliver genes into CD8 T cells (Zhou *et al*, 2015; Bender *et al*, 2016). Injecting these vectors once into “humanized” mice was able to generate CD19‐specific CAR‐T cells in 7 out of 10 mice. These CAR‐T cells successfully depleted human B cells. The “authenticity” of these cells is supported by the fact that three mice developed high levels of human cytokines and symptoms reminiscent of cytokine release syndrome, a well‐known problem in CAR‐T cell therapy that can be dealt with by using anti‐cytokine antibodies.

In the early days of monoclonal antibodies, the immunogenicity of murine monoclonal antibodies was envisaged as a major hurdle, leading to failure of the inventors' institution to patent the process. This hurdle was overcome in large part by appropriate genetic and molecular engineering, facilitating the success of immunotherapy to cytokines and cancers. In the present study, the benefit of combining basic research to improve the understanding of key molecules such as TcR, together with antibodies and genetic engineering skills to yield variants to a new clinically successful therapy, is clearly demonstrated. It will take considerable time and effort for the simplified process reported by Pfeiffer *et al* (2018) to be developed and able to reach patients, but it constitutes the first step towards converting CAR‐T cell therapy from an autologous, individualized to an off‐the‐shelf product, not only restricted to a lucky few.

## Conflict of interest

M. F. is a consultant to Celgene, Inc.
